# A Miniaturized Design for a Terahertz Tri-Mirror CATR with High QZ Characteristics

**DOI:** 10.3390/s25123751

**Published:** 2025-06-15

**Authors:** Zhi Li, Yuan Yao, Haiming Xin, Daocai Xiang

**Affiliations:** 1 EMC Research Center, China Electronics Standardization Institute, Beijing 100007, China; byliz@bupt.cn (Z.L.); xiangdc@cesi.cn (D.X.); 2 School of Electronic Engineering, Beijing University of Posts and Telecommunications, Beijing 100876, China; 3 Qilian Microwave Technology Co., Ltd., Chengdu 610095, China

**Keywords:** terahertz, shaped mirror, low cross-polarization, compact antenna test range

## Abstract

This paper proposes a miniaturized design for a terahertz tri-mirror compact antenna test range (CATR) system, composed of a square-aperture paraboloid primary mirror with a side length of 0.2 m and two shaped mirrors with circular apertures of 0.06 m and 0.07 m in diameter. The design first employs the cross-polarization cancelation method based on beam mode expansion to determine the geometric configuration of the system, thereby enabling the structure to exhibit low cross-polarization characteristics. Subsequently, the shaped mirrors, with beamforming and wave-front control capabilities, are synthesized using dynamic ray tracing based on geometric optics (GO) and the dual-paraboloid expansion method. Finally, the strong edge diffraction effects induced by the small-aperture primary mirror are suppressed by optimizing the desired quiet-zone (QZ) field width, adjusting the feed-edge taper, and incorporating rolled-edge structures on the primary mirror. Numerical simulation results indicate that within the 100–500 GHz frequency band, the system’s cross-polarization level is below −40 dB, while the amplitude and phase ripples of the co-polarization in the QZ are, respectively, less than 1.6 dB and 10°, and the QZ usage ratio exceeds 70%. The designed CATR was manufactured and tested. The results show that at 183 GHz and 275 GHz, the measured co-polarization amplitude and phase ripples in the system’s QZ are within 1.8 dB and 15°, respectively. While these values deviate slightly from simulations, they still meet the CATR evaluation criteria, which specify QZ co-polarization amplitude ripple < 2 dB and phase ripple < 20°. The overall physical structure sizes of the system are 0.61 m × 0.2 m × 0.66 m. The proposed miniaturized terahertz tri-mirror CATR design methodology not only enhances the QZ characteristics but also significantly reduces the spatial footprint of the entire system, demonstrating significant potential for practical engineering applications.

## 1. Introduction

With the rapid development of terahertz technology, the types of terahertz antennas have become increasingly diverse [1,2,3,4,5]. As an important means of accurately measuring the radiation characteristics of antennas, CATR systems not only generate quasi-planar waves within a confined space to meet measurement requirements but also eliminate the need for long distances inherent in far-field testing [6,7]. These capabilities have driven increasing demand for CATR systems in evaluating the performance of various terahertz antennas. At present, most CATR systems feature large apertures and significant space requirements, leading to high construction costs. However, when testing small-scale terahertz antenna devices such as those used in 5G/6G high-speed communication systems, vehicle-mounted radar, and low-Earth orbit (LEO) satellite terminals, the available space for the entire measurement system is often very limited [8,9,10]. Therefore, to meet the test requirements of high-frequency, small-scale antennas under test (AUTs) while reducing construction costs and the space occupation of the measurement system, there is an urgent need to design and develop a miniaturized, low-cost, high-performance CATR system with integrated and flexible mobility configurations.

Currently, based on the number of mirrors, mirror CATRs are mainly divided into three configurations: single-mirror, dual-mirror, and tri-mirror CATRs [11,12,13,14]. Single-mirror CATRs, incapable of beam conversion and shaping, generally exhibit a low QZ usage ratio (typically below 50%) and suffer from higher cross-polarization levels due to inherent offset structural distortions [15]. In dual-mirror CATRs, the introduced mirror, which is comparable in size to the primary mirror, not only greatly increases the edge diffraction effects of the mirrors, leading to significant degradation in system performance, but also increases construction costs [16]. In contrast, tri-mirror CATR systems, incorporating two small shaped mirrors, demonstrate distinct advantages in both performance and cost-effectiveness. These shaped mirrors can redistribute the energy of the electromagnetic beam radiated by the feed source into the desired outgoing field, thereby achieving better QZ characteristics than single- and dual-mirror CATRs. Furthermore, the mirrors’ dimensions are substantially smaller than those of the primary mirror, drastically reducing fabrication costs and manufacturing cycles.

However, current research and development for integrated, miniaturized CATR systems are predominantly focused on low-frequency, single-mirror configurations [17,18]. Examples include Keysight’s F9650A (Keysight Technologies, Inc., Santa Rosa, CA, USA) miniaturized single-mirror CATR [19], with an overall footprint of 2.8 m × 1.6 m × 2 m and a primary mirror size of 0.6 m × 0.6 m, achieving a QZ usage ratio of 50%. Other notable CATR systems include General Test’s RayPact600C (General Test Systems Inc., Shenzhen, China) series [20], C&G Microwave’s CATR-30R (C&G Microwave Co., LTD., Daejeon, South Korea) [21], Guoyu Microwave’s D0303R (Guoyu Microwave, Beijing, China) [22], and NSI-MI’s CRR-CSC-0.3 (NSI-MI Technologies, Suwanee, GA, USA) miniaturized single-mirror CATRs [23]. Although these CATR systems can provide a QZ of approximately 0.3 m × 0.3 m within a relatively small space, with a QZ usage ratio of 50%, there has been no significant research on miniaturized terahertz tri-mirror CATRs, which are expected to offer superior QZ characteristics, particularly in terms of cross-polarization suppression. Furthermore, although the above-mentioned miniaturized single-mirror CATRs can meet general testing requirements, they still face the following challenges: (1) a relatively low operating frequency and limited frequency bandwidth, (2) a low QZ usage ratio and small QZ size, and (3) high cross-polarization levels.

To address the above challenges, this paper presents a miniaturized terahertz tri-mirror CATR design methodology with low cross-polarization, operating in the 100–500 GHz band. Based on the large-aperture CATR design in [24,25], this design proposes a method using the beam mode expansion technique, regulating the expected output field QZ radius and the edge taper of the feed, and incorporating rolled-edge structures on the primary mirror to achieve a high-performance CATR design with a small aperture, low edge diffraction, and low cross-polarization. Meanwhile, during experimental measurements, this work references content from [26,27,28] on planar wave area error analysis, mirror surface roughness effects, and the application of microwave-absorbing materials on the test prototype surface for a sub-THz CATR, thereby reducing measurement inaccuracies in the system prototype. Specifically, the methodology first determines the geometric configuration of the tri-mirror CATR system under low cross-polarization constraints by using cross-polarization cancelation conditions based on beam mode analysis theory [29]. Then, building on dynamic ray tracing based on GO and the dual-paraboloid expansion method [30,31], the two shaped mirrors are shaped by adjusting the expected output near-field QZ width and feed-edge taper. Finally, a rolled-edge treatment is applied to the paraboloid primary mirror to suppress edge diffraction effects [32], achieving high QZ characteristics. Following this approach, a miniaturized tri-mirror CATR system with low cross-polarization is designed, featuring a small main structure size of 0.61 m × 0.2 m × 0.66 m. The results of the numerical simulations and experimental testing demonstrate excellent agreement, validating the correctness and effectiveness of the proposed methodology. This design approach provides a theoretical foundation and technical support for designing and constructing flexible, portable, and high-performance miniaturized CATR systems in spatially constrained measurement environments.

## 2. Design of a Miniaturized Tri-Mirror CATR

In order to design a miniaturized tri-mirror CATR with low cross-polarization and high QZ performance, two critical issues need to be solved. The first is the determination of the geometric structure configuration for the miniaturized tri-mirror CATR under low cross-polarization conditions, and the second is the shaping design and synthesis of the two shaped sub-mirrors of this CATR system. For these two critical issues, Section 2.1 and Section 2.2, respectively, provide specific solutions and detailed design procedures.

### 2.1. Cross-Polarization Cancelation Method and Geometric Configuration

**Figure 1:**
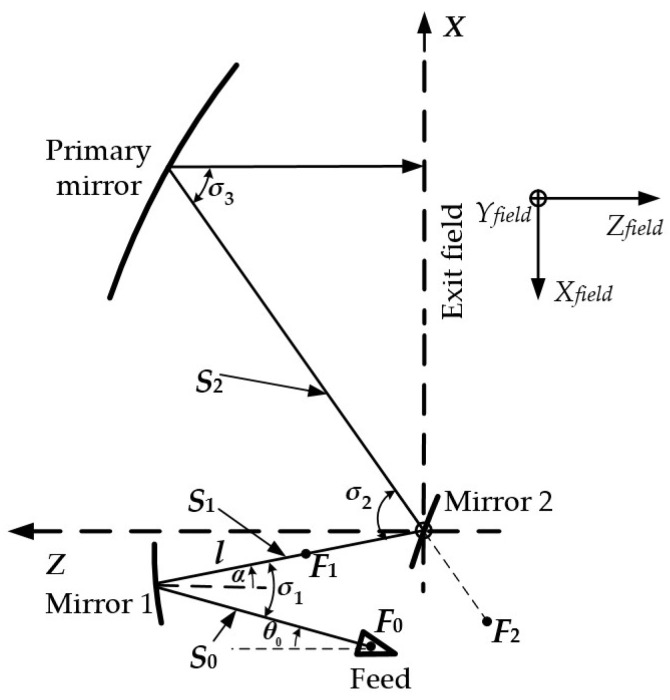
Schematic diagram and parameter variables of the tri-mirror system.

For a tri-mirror CATR, the geometric positions of all the mirrors have a significant influence on the system’s cross-polarization. Therefore, to realize low cross-polarization for a miniaturized tri-mirror CATR, it is essential to first determine the geometric layout of the system under low cross-polarization constraints and calculate precisely each reflector’s center coordinates. As illustrated in Figure 1, the developed tri-mirror system comprises a feed, two small mirrors, and a paraboloid primary mirror. Specifically, mirror 1 is configured with confocal foci at *F*_0_ and *F*_1_, while mirror 2 shares confocal foci at *F*_1_ and *F*_2_, and the paraboloid primary mirror has its focal point at *F*_2_. The equivalent focal lengths of mirror 1, mirror 2, and the primary mirror are denoted *f*_1_, *f*_2_, and *f*_3_, respectively. In this system, cross-polarization cancelation conditions based on beam mode expansion theory have been adopted for the system’s structural configuration design and the calculation of optical center coordinates in each mirror.

In order to enable the tri-mirror system to achieve a low cross-polarization structural layout, according to the definition of the equivalent focal length of the mirrors in multi-mirror systems and the derivation of the low cross-polarization formula in [29], the parameter variables of the designed tri-mirror system illustrated in Figure 1 should satisfy the following equations:

(1) $$\frac{s_{2}tan(\sigma_{3}/2)}{f_{3}\cdot s_{1}tan(\sigma_{1}/2)}=\frac{1}{s_{0}}+\frac{1}{l}$$

(2) $$\frac{1/s_{1}+1/s_{2}}{\left\{f_{3}tan(\sigma_{2}/2)/s_{1}tan(\sigma_{3}/2)\right\}-1}=\frac{1}{f_{3}-s_{2}}-\frac{1}{s_{1}-l}$$

(3) $$f_{2}=\frac{\left\{f_{3}tan(\sigma_{2}/2)/s_{2}tan(\sigma_{3}/2)\right\}-1}{1/s_{1}+1/s_{2}}$$

where variable *l* denotes the distance between the optical center of mirror 1 and the confocal focus, *F*_1_. *S_i_* (*i* = 1, 2, 3) denotes the distance of the central beam in each of the three propagation segments as rays emitted from the feed sequentially pass through mirror 1, mirror 2, and the primary mirror. *σ_i_* (*i* = 1, 2, 3) is denoted as the angle formed by the incident central beam and the reflected central beam of each mirror. *α* is the angle formed between the propagation direction of the central reflected beam of mirror 1 and the horizontal axis direction, with *α* defined as positive in the counterclockwise direction. *θ*_0_ is the angle formed between the radiation direction of the central beam of the feed and the horizontal axis direction. All the above variables are marked in the system depicted in Figure 1.

Next, the value relationships of the above-mentioned structural parameter variables will be analyzed and solved, and the geometric layout of the tri-mirror system will be determined. Guided by the principles of miniaturization and practicality, the primary mirror and mirror 1 in the tri-mirror system will be assumed to both be concave mirrors. According to the cross-polarization cancelation condition based on the beam mode analysis method and the system’s structural definition in [29], it can be seen that *f*_1_ > 0, *f*_3_ > 0. Additionally, when the central ray between mirror 1 and mirror 2 lies above the horizontal axis passing through the optical center of mirror 1 (i.e., *α* > 0), the following conditions hold: *σ*_2_ > *σ*_3_, *f*_3_ > *S*_2_. Thus, the following relationship can be derived:

(4) $$\frac{f_{3}}{s_{2}}>\frac{tan(\sigma_{3}/2)}{tan(\sigma_{2}/2)}$$

Then, substituting Equation (4) into Equation (3) yields *f*_2_ < 0; as stated in [29], mirror 2 is a convex mirror. Under these parameter constraints, the resulting geometric layout of the tri-mirror system will exhibit a state of low cross-polarization, as illustrated in Figure 1.

To determine the optical center positions of the mirrors in the low cross-polarization geometric configuration, a global coordinate system, *XYZ*, with the optical center of mirror 2 as the origin is established. First, the relevant structural parameters of the system are substituted into Equations (1) and (2), satisfying the cross-polarization cancelation conditions. Next, based on design requirements, the parameter variables *θ*_0_ and *S*_0_ are assumed to be the desired constant values. Here, *θ*_0_ is set to 15°, and *S*_0_ = 0.13 m is substituted into Equation (1). Then, Equations (1) and (2) are programmed and solved in the numerical computation software MATLAB R2018b. By using the values of the previously known variables, *α*, *σ*_2_, *S*_1_, and *f*_3_, the values of the remaining key unknown variables, *S*_2_, *l*, *σ*_1_, and *σ*_3_, can be obtained, and the coordinates of the optical centers of each mirror can be further derived. Table 1 shows the key parameter values of the tri-mirror system with low cross-polarization. This process completes the determination of the geometric configuration and optical center positions for the low cross-polarization tri-mirror system.

### 2.2. Synthesis of Shaped Mirrors and System Design

After determining the geometric layout of the tri-mirror CATR and the optical center coordinates of each mirror, dynamic ray tracing based on GO and the dual-paraboloid expansion method in [31] are employed to synthesize the two shaped mirrors of the system. The shaped mirror synthesis process adheres to the principles of energy conservation and equal optical path. Dividing the spherical beam radiated from the feed source into an infinite number of sub-beams establishes a one-to-one correspondence with countless discrete target points in the desired exit QZ field of radius, *r*, while redistributing energy. The entire beam-tracing process employs the double paraboloid expansion method, starting with the central beam emitted from the feed source, which sequentially expands outward along the optical central points of each mirror and undergoes beam rearrangement until the final sub-beam is traced. The innumerable sub-beams intersect the spatial surfaces containing the optical centers of the two shaped mirrors, forming densely packed and smoothly connected discrete points that collectively compose the shaped mirror surfaces. Following the design procedure described above, a mapping function (*x*, *y*) = *F*(*θ*, *φ*) between the beam with the output direction (*θ*, *φ*) emitted from the feed and the position (*x*, *y*) where the desired output field is established using the dynamic ray tracing method [31]. This process is programmed and numerically computed in the numerical software MATLAB R2018b to generate numerous discrete data points representing the two shaped mirrors. The data are then imported into the electromagnetic simulation software GRASP-10, which is based on physical optics and the physical theory of diffraction, for modeling and simulation. The combination of these two shaped mirrors and the paraboloid primary mirror can significantly improve the QZ characteristics of the system. Furthermore, analysis of the designed feed and the desired output QZ field reveals that they are two critical factors influencing the system’s QZ characteristics. For the horn feed, its normalized far-field is represented by

(5) $$G(\theta )=10^{\left[\frac{T_{i}}{20}{\left(\frac{\theta }{\theta_{1}}\right)}^{2}\right]}$$

where *θ* is the angle of the output field relative to the boresight direction of the feed, and *T*_i_ represents the edge taper of the feed at a specific angle, *θ*_1_. For a fixed feed flare-angle, a larger edge taper results in a steeper drop-off in the far-field distribution, leading to more concentrated beam energy radiated by the feed and reduced diffraction effects in the system. However, higher edge taper values increase the fabrication complexity of the feed. Therefore, in high-performance CATR designs, the edge taper can be treated as an optimization variable to balance performance and manufacturability.

For the expected output QZ field, its field strength distribution can be expressed as

(6) $$E(r)=\left\{\begin{array}{l} 1\;,\;\;\;\;\;\;\;\;\;\;\;\;\;\;\;\;\;\;\;\;\;\;\;\;\;\;\;\;\;r_{q}\geq r\geq 0\\ 10^{\frac{T_{0}}{20}{\left(\frac{r-r_{q}}{r_{\max}-r_{q}}\right)}^{2}},\;r_{\max}\geq r>r_{q} \end{array}\right.$$

where *r* is the radius of the output field; *r*_q_ is the expected QZ radius; *r*_max_ represents the radius of the system’s primary mirror; and *T*_0_ is the expected edge taper of the output field at *r*_max_. The expected output QZ field features a flat distribution in the center region and Gaussian steep drop-offs at both edges. For such a field distribution, when the system’s primary mirror radius, *r*_max_, is fixed, the QZ width of the output field can be increased by appropriately enlarging the expected QZ radius, *r*_q_. Consequently, the parameter *r*_q_ is also treated as an optimization variable for enhancing QZ characteristics.

**Figure 2:**
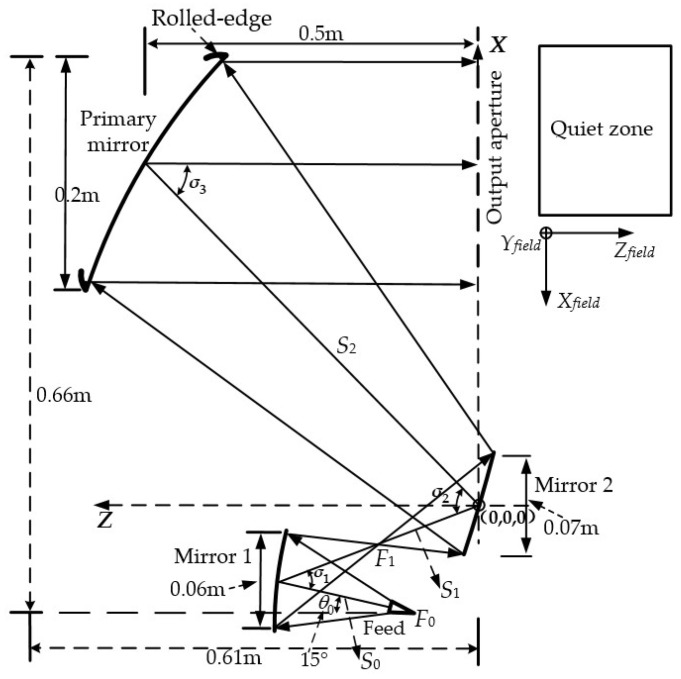
Configuration structure of the designed miniaturized tri-mirror CATR with low cross-polarization.

For the primary mirror, as its aperture size decreases, the edge diffraction effects of the mirror increase sharply. Simultaneously, to improve the system’s QZ usage ratio, the method of increasing the output field QZ width adopted during the shaped mirror design also results in a relatively divergent energy distribution at the primary mirror’s edges, further exacerbating edge diffraction. Therefore, to reduce the impact of edge diffraction on the system’s QZ, the rolled-edge treatment in [32] is applied to the edge of the primary mirror in the tri-mirror CATR system. Following the above design process, a miniaturized tri-mirror CATR system working at 100–500 GHz with an aperture of 0.2 m is developed. Its configuration structure is illustrated in Figure 2, and the main structural dimensions of the entire system are 0.61 m × 0.2 m × 0.66 m.

## 3. Simulation and Measurement Results

**Figure 3:**
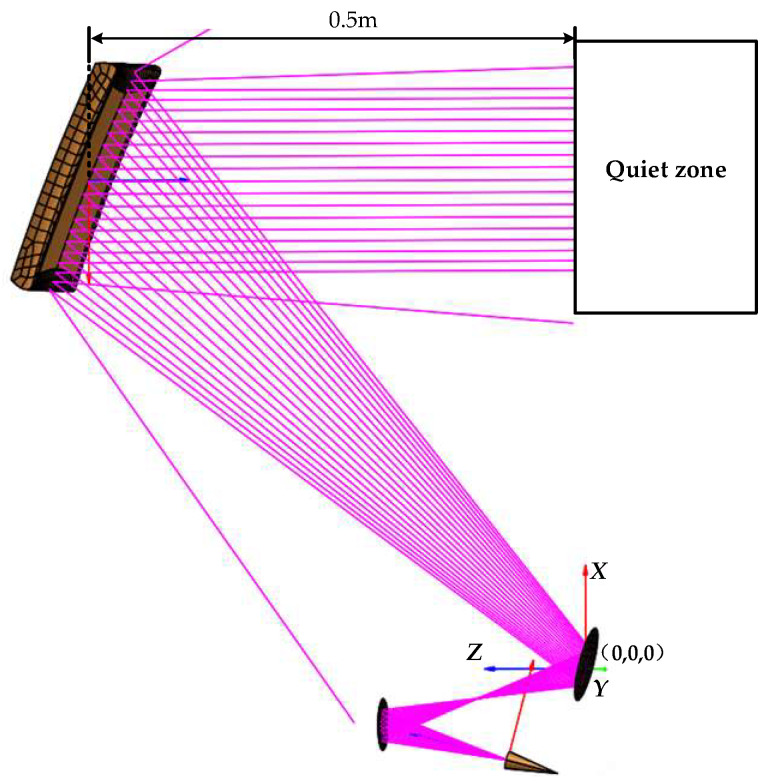
Simulation model for the developed miniaturized tri-mirror CATR with low cross-polarization.

To validate the QZ characteristics of the designed miniaturized tri-mirror CATR system, numerical simulations and prototype testing of the system are conducted in this section. Figure 3 illustrates the simulation model of the designed system, which is numerically analyzed using the electromagnetic simulation software GRASP-10 based on Physical Optics and Theory of Diffraction algorithms across the 100–500 GHz operating frequency band. During the simulation modeling, all three mirrors are assumed to be made of PEC (Perfect Electric Conductor) material with ideal conductive properties, and the surroundings of the model are set to an ideal environment without reflected waves. In the simulation, the system employs an ideal Gaussian feed with a cross-polarization amplitude under −50 dB, a far-field pattern featuring good symmetry characteristics, and a pattern taper with −16 dB attenuation at 15°.

**Figure 4:**
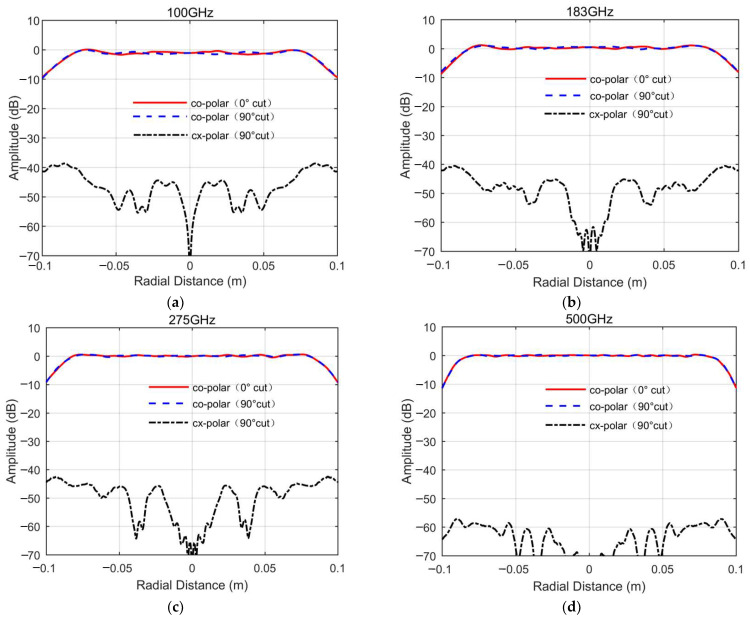
The simulated QZ field cross-polarization amplitude curves of the designed miniaturized tri-mirror CATR system: (**a**) 100 GHz; (**b**) 183 GHz; (**c**) 275 GHz; (**d**) 500 GHz.

**Figure 5:**
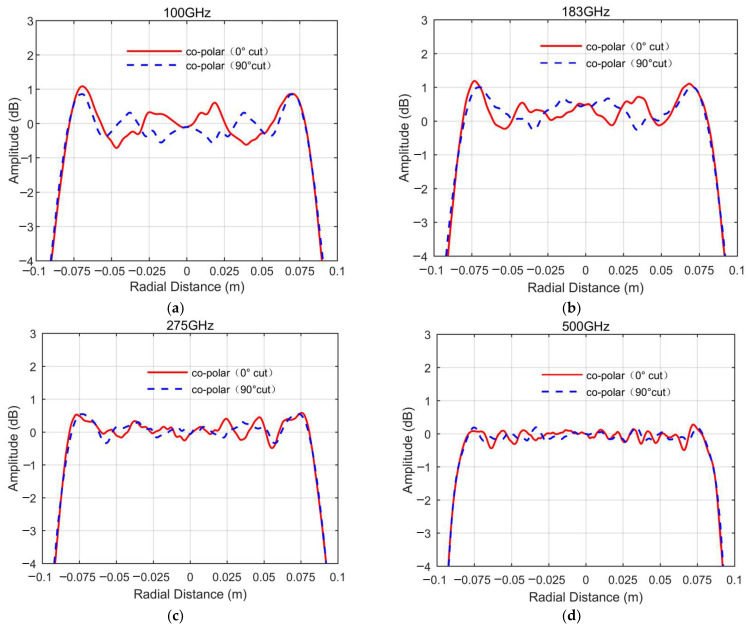
The simulated QZ field co-polarization amplitude curves of the designed miniaturized tri-mirror CATR system: (**a**) 100 GHz; (**b**) 183 GHz; (**c**) 275 GHz; (**d**) 500 GHz.

**Figure 6:**
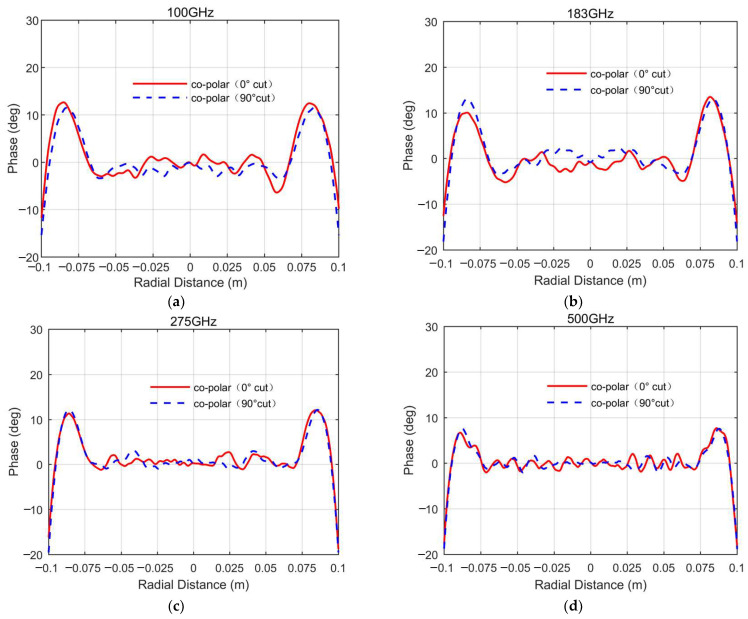
The simulated QZ field co-polarization phase curves of the designed miniaturized tri-mirror CATR system: (**a**) 100 GHz; (**b**) 183 GHz; (**c**) 275 GHz; (**d**) 500 GHz.

Figure 4, Figure 5 and Figure 6, respectively, show the simulated QZ field distribution results for the major cuts of the horizontal and vertical planes for the developed miniaturized tri-mirror CATR system at 100 GHz, 183 GHz, 275 GHz, and 500 GHz.

The simulated outcomes above clearly demonstrate that the tri-mirror CATR achieves co-polar amplitude and phase ripples of 1.6 dB and 10°, respectively, with all cross-polarizations under −40 dB across the major cuts of the QZ in a frequency range of 100–500 GHz. Notwithstanding the presence of undesirable ripples in the central QZ region arising from mirror-edge diffraction effects and the multi-path scattering of stray fields, particularly pronounced at lower frequencies, the co-polarization amplitude and phase profiles of the system’s QZ remain sufficiently stable across the operational bandwidth. Analysis of the simulation data further reveals a well-defined QZ diameter of 0.14 m for both co-polar amplitude and phase characteristics in the central region, corresponding to a high QZ utilization ratio of 70%. This outcome validates the superior QZ characteristics of the engineered miniaturized tri-mirror CATR system. The observed disturbances are primarily attributable to edge diffraction phenomena and recirculating wave interactions within the chamber environment, yet the fundamental field uniformity remains preserved across the operational bandwidth. Table 2 summarizes the simulation data of the QZ characteristics of the major cuts of the horizontal and vertical planes within the 0.14 m QZ diameter range for the designed miniaturized tri-mirror CATR.

Analysis of the simulation data in Table 2 demonstrates that the engineered tri-mirror CATR achieves broadband operation across 100–500 GHz. At lower frequencies, the co-polarization amplitude and phase ripple values are elevated due to the combined effects of mirror edge diffraction and surface roughness. However, as the operating frequency increases, these perturbations attenuate monotonically, leading to progressive improvement in QZ stability. This frequency-dependent behavior highlights the system’s ability to maintain acceptable QZ performance across an extended bandwidth.

**Figure 7:**
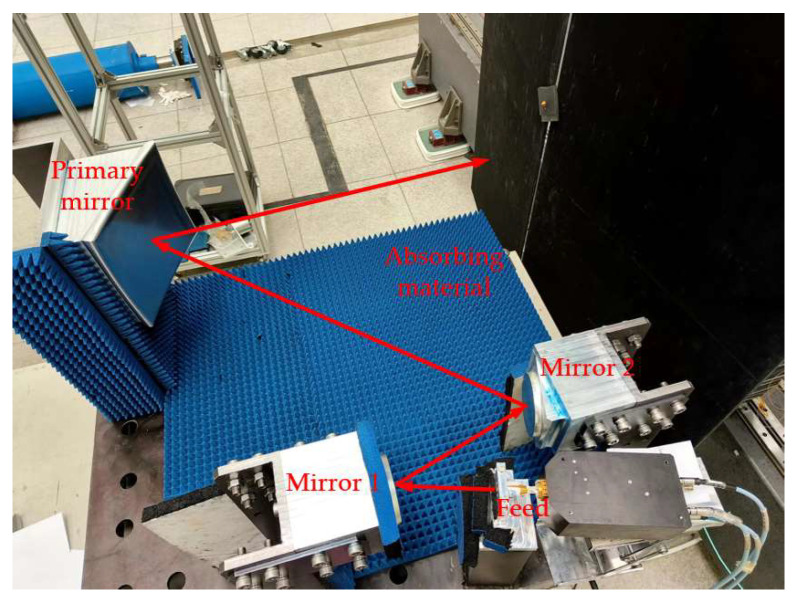
The physical prototype of the fabricated miniaturized tri-mirror CATR system.

Although the simulations offer valuable insights into the system’s performance, several limitations need to be acknowledged. First, the simulations employed an idealized feed model with a cross-polarization of less than −50 dB, which contrasts with practical feeds like corrugated horns that show higher cross-polarization, reaching −29 dB during testing. Second, perfect electrical conductor (PEC) boundary conditions were applied to all mirrors and fixtures. However, the actual surface roughness (with an RMS of approximately 12 µm) and the finite conductivity of aluminum/stainless steel materials were not considered in the model, potentially resulting in underestimated losses and edge diffraction. Third, the electromagnetic (EM) model omitted brackets and fixtures, and while their scattering effects were partially reduced experimentally using absorbers (as shown in Figure 7), this simplification still affects the accuracy. Fourth, at lower frequencies below 200 GHz, the Physical Optics and Theory of Diffraction algorithms may fail to adequately model complex multi-path interactions. Finally, the simulations assumed an ideal alignment, while the prototype assembly introduced positional errors of around 20 µm and phase center misalignments, further deviating from the idealized simulation conditions.

To further validate the effectiveness of the proposed design methodology, the miniaturized tri-mirror CATR system was fabricated and experimentally tested. The simulation model shown in Figure 3 was positioned in a horizontal orientation, with the three mirrors and feed fixed on a common planar substrate via brackets for stable support. The feed was secured using a replaceable ‘cap-style’ aluminum alloy module. The physical prototype of the fabricated miniaturized tri-mirror CATR system is shown in Figure 7.

All fixtures and planar substrates of the CATR prototype are fabricated from stainless steel, while the three mirrors are manufactured from aluminum alloy. Precision high-speed milling and mirror-polishing processes are applied to the aluminum alloy blocks, achieving a root mean square (RMS) surface accuracy of approximately 12 μm and a surface roughness of 1 μm for the main mirror. Meanwhile, the relative positional errors of the three mirrors during assembly are controlled within approximately 20 μm. During system testing, to reduce interference from stray waves reflected by the stainless-steel substrates and fixtures on the system’s QZ, a layer of microwave-absorbing material is applied to the surfaces of planar substrates and fixtures to partially absorb stray signals, as shown in Figure 7.

**Figure 8:**
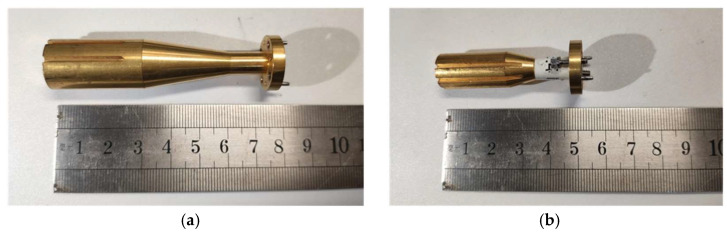
Manufactured physical prototype of corrugated horn feeds for the tri-mirror CATR: (**a**) with center operating frequency of 183 GHz; (**b**) with center operating frequency of 275 GHz.

The feed source for the system employs a laboratory-available sin-squared/horizontal linear dual-profile corrugated horn [33]. This horn feed features a highly symmetric radiation pattern, relatively low side-lobe levels, and a phase center located near the horn’s aperture plane at the center operating frequency, facilitating position fixation and alignment during actual testing [34]. Figure 8a and Figure 8b, respectively, show fabricated corrugated horn feeds with center operating frequencies of 183 GHz and 275 GHz; where the feeds taper, both satisfy −16 dB attenuation at 15°. These horns were manufactured using an electroforming method and underwent surface gold-plated treatment.

**Figure 9:**
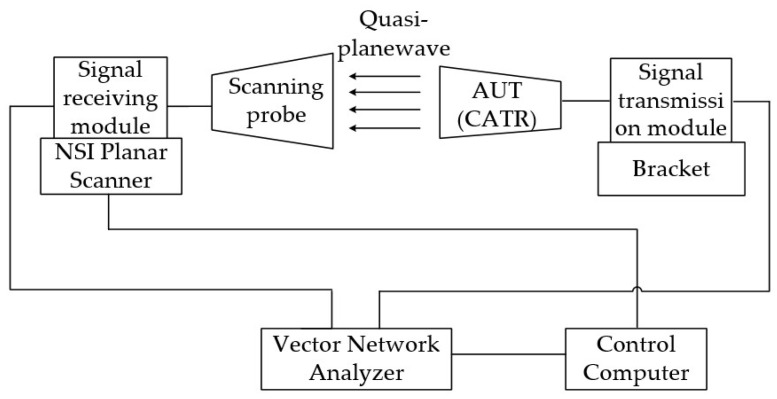
Schematic diagram of the planar near-field test for the QZ of the tri-mirror CATR.

After the fabrication and assembly of the designed tri-mirror CATR, the QZ generated by the prototype was scanned and performance-tested using the NSI planar near-field scanning system. Figure 9 shows the schematic diagram of the planar near-field test for the QZ of the designed tri-mirror CATR prototype. The entire testing procedure begins with the control computer issuing test commands. Subsequently, the built-in fundamental signal source of the Vector Network Analyzer N5242A (VNA) transmits signals, which are processed by a transmitting module incorporating a power amplifier and a frequency extender to generate terahertz signals across different frequency bands. These signals are then radiated as quasi-planar waves from the feed source toward the main reflector of the CATR under test via the transmitting module. Next, a signal-receiving scanning probe connected to the signal reception module on the NSI High-Precision Planar Near-Field Scanner scans and captures the field distribution within the QZ plane at a specified distance perpendicular to the quasi-planar waves. The received signals are modulated by a receiving module with a low-noise amplifier (LNA) and fed back into the VNA. Finally, through data processing and analysis, various QZ performance parameters are obtained. The entire testing process is controlled by the NSI-300V-12x12 software.

**Figure 10:**
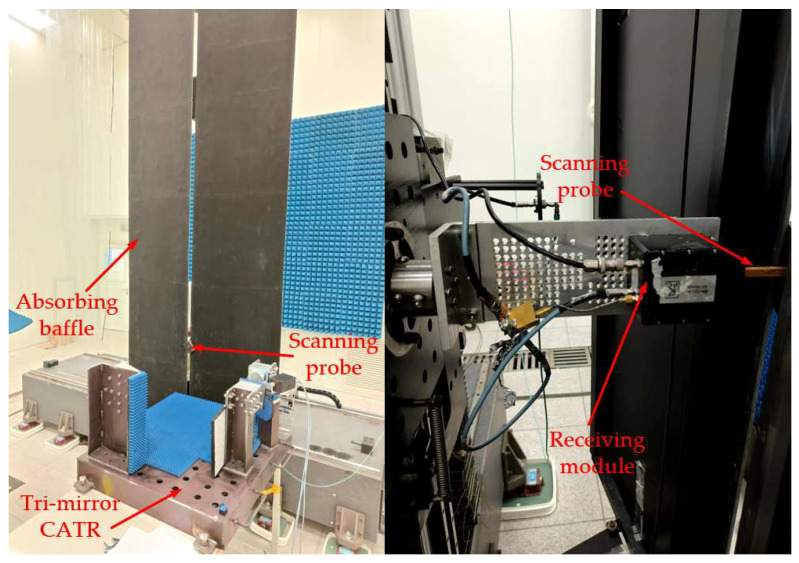
NSI planar near-field test scanner used for the designed tri-mirror CATR testing.

Figure 10 depicts the planar near-field testing environment for the designed tri-mirror CATR. The NSI planar near-field scanner features a scanning range of 2.4 m × 2.4 m, a flatness better than 5 μm, and a testing frequency up to 500 GHz. A vertical plane located 75 cm from the center of the primary mirror was selected as the QZ scanning plane, with a scanning area of 20 cm × 20 cm and a sampling grid of 101 × 101 points.

**Figure 11:**
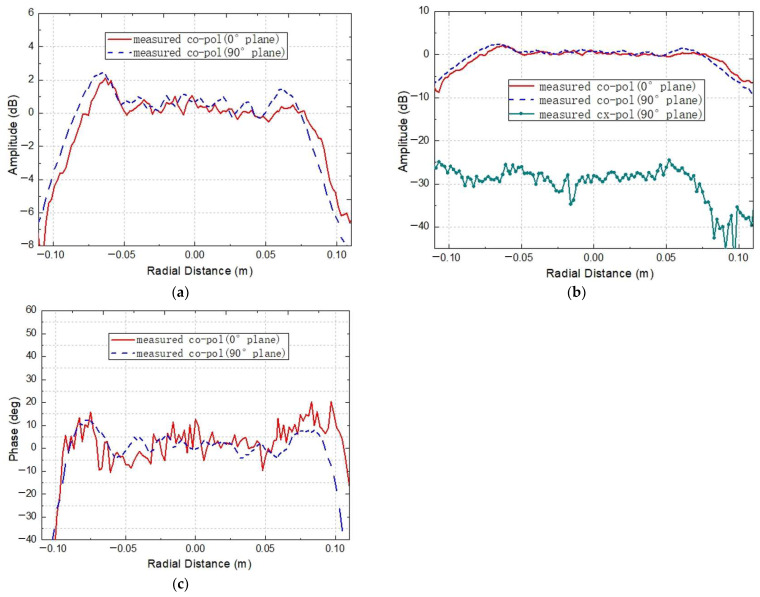
The measured QZ field distribution curves for the designed miniaturized tri-mirror CATR at 183 GHz: (**a**) the measured co-polarization amplitude distribution; (**b**) the measured cross-polarization amplitude distribution; (**c**) the measured co-polarization phase distribution.

**Figure 12:**
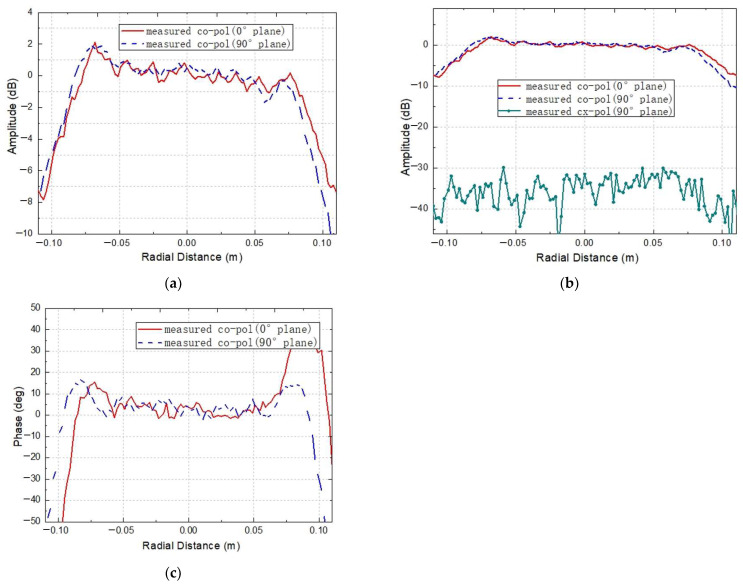
The measured QZ field distribution curves for the designed miniaturized tri-mirror CATR at 275 GHz: (**a**) the measured co-polarization amplitude distribution; (**b**) the measured cross-polarization amplitude distribution; (**c**) the measured co-polarization phase distribution.

For the developed tri-mirror CATR, its operating frequency covers multiple frequency bands, while the bandwidth of a single horn feed is relatively limited. Thus, measurements across the full frequency range of the system can be achieved by replacing the horn feeds and corresponding frequency extension modules for different bands. Constrained by the frequency extension modules available on the near-field testing platform, two corrugated horns with central operating frequencies of 183 GHz and 275 GHz, as shown in Figure 8, are used to feed the miniaturized tri-mirror CATR system. Figure 11 and Figure 12 present the measured QZ field distribution curves of the co-polarization amplitude/phase and cross-polarization amplitude of the major cuts of the horizontal and vertical planes at 183 GHz and 275 GHz, respectively.

As shown in Figure 11 and Figure 12, the measured QZ results at 183 GHz and 275 GHz indicate that the co-polarization amplitude ripples within the QZ of the CATR system are all within 1.8 dB. The co-polarization phase ripples are all within 11°, except for significant fluctuations in the horizontal direction at 183 GHz, and the QZ usage ratio reaches 70%. Additionally, the co-polarization amplitude and phase of the QZ field in the central region exhibit overall flat distributions, while rapid attenuation occurs on both edges, which is consistent with the previous simulation results of the system. The pronounced phase fluctuations in the horizontal direction at 183 GHz can be attributed to misalignment errors in the feed’s phase center position and the presence of scattering objects near the system’s lateral sides during testing. Although the experimental results at both frequencies are slightly higher than the simulation results, the measured performance remains satisfactory considering fabrication tolerances, calibration uncertainties, and measurement errors, meeting the general requirements for terahertz-band antenna testing. Table 3 lists the measured results of the designed miniaturized tri-mirror CATR at 183 GHz and 275 GHz, respectively.

**Figure 13:**
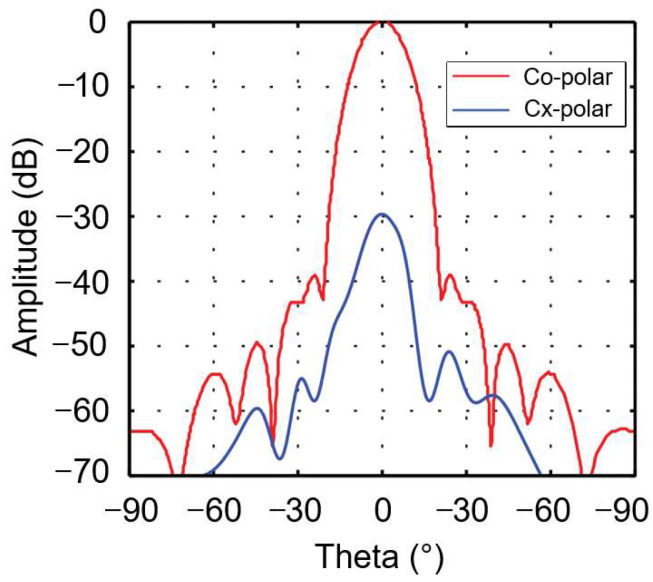
The experimental far-field pattern of the corrugated horn feed with a center operating frequency of 183 GHz.

When comparing the measurement results in Table 3 with the simulation results in Table 2, it can be seen that the measured cross-polarization levels of the CATR system at 183 GHz and 275 GHz are −25.3 dB and −32.2 dB, respectively, deviating significantly from the simulated results (−44.5 dB). This discrepancy arises primarily from three interrelated factors: Firstly, the inherent cross-polarization of the corrugated horn (−29 dB, as characterized in Figure 13) directly compromised system performance. Re-simulation using the horn’s measured radiation pattern (detailed in Table 4) confirmed this effect, yielding a cross-polarization value of −28.1 dB—closely aligned with the measured −25.3 dB, which highlights the feed’s critical role in degrading performance. Secondly, stray-field interactions occurred: Reflections from stainless-steel fixtures—only partially mitigated by absorbers during testing (as shown in Figure 7)—and scattering objects near the test setup contributed to horizontal phase fluctuations (e.g., 23.5° at 183 GHz). These non-ideal electromagnetic couplings were not fully captured in the simulation model, contributing to residual discrepancies. Thirdly, fabrication and alignment tolerances also contributed: Surface roughness (1 µm RMS), mirror positional errors (~20 µm), and feed-phase-center misalignment exacerbated ripples, especially at higher frequencies. These mechanical imperfections, while small in absolute terms, became more pronounced in the narrowband high-frequency regime. Despite these discrepancies, co-polarization amplitude (≤1.8 dB ripple) and QZ utilization (70%) aligned well with the simulations, validating the core design methodology. The experimental far-field radiation pattern of the 183 GHz corrugated horn feed shown in Figure 8a is presented in Figure 13.

**Figure 14:**
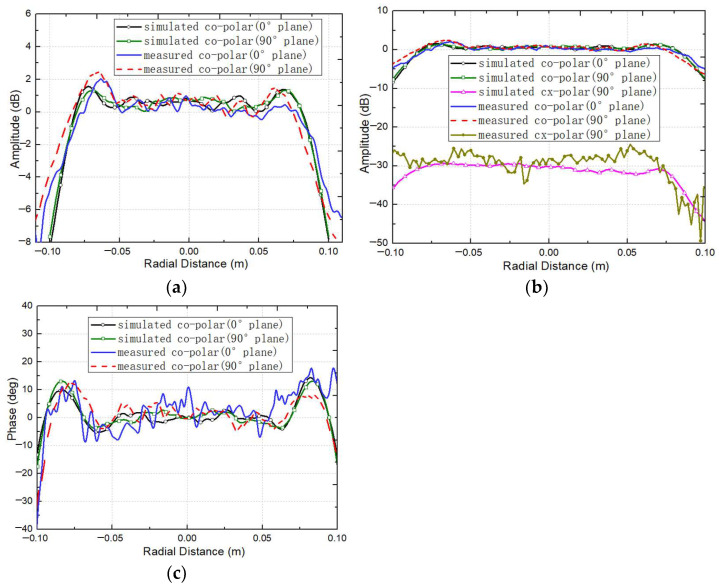
The simulated and measured QZ field distribution curves of the system when fed by the corrugated horn feed with a center operating frequency of 183 GHz: (**a**) the co-polarization amplitude of the QZ field; (**b**) the cross-polarization amplitude of the QZ field; (**c**) the co-polarization phase of the QZ field.

As can be seen from Figure 13, the corrugated horn feed exhibits excellent symmetry in co-polarization amplitude and meets the taper requirement of −16 dB attenuation at 15°, with side-lobe levels below −40 dB. However, its cross-polarization amplitude is only −29 dB. Since this cross-polarization amplitude is significantly higher than that of the ideal Gaussian feed (cross-polarization amplitude < −50 dB) used in previous CATR simulations, feeding the designed CATR system with this corrugated horn feed during testing will inevitably result in higher measured cross-polarization levels in the system’s QZ compared to the simulated results. To verify this inference and accurately evaluate the cross-polarization performance of the designed CATR system, the measured radiation pattern data of the corrugated horn feed with the center operating frequency of 183 GHz, shown in Figure 13, were substituted for the data of the ideal Gaussian feed pattern used in the previous simulations. The designed tri-mirror CATR system was then re-simulated at 183 GHz, and the newly obtained simulation results were compared with the system’s measured results to validate the correctness of the above inference. Figure 14 shows the simulated and measured QZ field distribution curves of the system when fed by the corrugated horn feed with a center operating frequency of 183 GHz.

According to the simulation results in Figure 14, when the CATR system is fed by the corrugated horn with a center operating frequency of 183 GHz, the simulated QZ co-polarization amplitude and phase ripples are 1.4 dB and 8.3°, respectively, with a cross-polarization amplitude of −28.1 dB. The measured results show corresponding values of 1.64 dB, 10.1°, and −25.3 dB, demonstrating excellent agreement between the simulation results and the measured results. Furthermore, comparing the simulated QZ characteristics using the corrugated horn feed (cross-polarization amplitude: −29 dB) in Figure 14b with that using the ideal Gaussian feed (cross-polarization amplitude < −50 dB) in Figure 4b reveals that the system’s other QZ characteristics metrics (e.g., amplitude/phase uniformity) remain essentially consistent, except for the significant deviation in cross-polarization. This confirms that the elevated cross-polarization levels in the measured results are directly attributable to the inherent higher cross-polarization of the corrugated horn feed. Table 4 summarizes the simulated QZ results with the ideal Gaussian feed (cross-polarization amplitude < −50 dB), the measured QZ results with the corrugated horn feed (cross-polarization amplitude: −29 dB), and the re-simulated QZ results using the corrugated horn’s measured pattern (cross-polarization amplitude: −29 dB) at 183 GHz.

According to the experimental results in Table 4, when the system is fed by feeds with identical taper profiles but different cross-polarization characteristics, the co-polarization amplitude and phase in the QZ remain consistent in the above three different cases. Minor deviations (<0.3 dB/2.6° in co-polarization) between re-simulation and measurement arise from un-modeled test-environment effects (e.g., stray waves and calibration uncertainties). Regarding cross-polarization, the measured QZ cross-polarization amplitude of the designed CATR system deviates significantly from the simulated results obtained with the ideal Gaussian feed (cross-polarization amplitude: < −50). However, when the corrugated horn feed (cross-polarization amplitude: −29 dB) is used in simulations, the simulated cross-polarization amplitude (−28.1 dB) differs from the measured value (−25.3 dB) by only 2.8 dB, demonstrating close alignment between the re-simulation results (using measured feed patterns) and the measured results. This not only fully confirms that feed non-ideality is the dominant factor in the cross-polarization discrepancies of the CATR system but also validates the effectiveness of the proposed miniaturized tri-mirror CATR design methodology in achieving low cross-polarization performance.

Finally, Table 5 lists the major characteristics between the proposed and the already-reported miniaturized CATR. As shown in Table 5, although the CATR in [19,20,21,22,23] achieves 50% QZ utilization in a relatively small space with only one mirror, our proposed miniaturized tri-mirror CATR composed of three mirrors exhibits higher QZ utilization (up to 70%), a higher upper frequency limit, lower cross-polarization (below −40 dB), and a smaller system size. This miniaturized tri-mirror CATR design approach paves a new path for overcoming and reducing the high edge diffraction effects in small-aperture CATRs, as well as for constructing high-performance, flexible-mobility CATRs under constrained site space conditions.

## 4. Conclusions

In this paper, a design methodology for a miniaturized tri-mirror CATR system with high QZ characteristics is proposed. By employing cross-polarization cancelation conditions based on beam mode analysis theory, dynamic ray tracing based on geometric optics, shaped mirror surface reconstruction techniques, and a method of adding rolled-edge structures on the primary mirror, a miniaturized tri-mirror CATR system operating in the 100–500 GHz band is designed and numerically simulated. The system features a primary mirror with a 0.2 m diameter and two shaped mirrors with diameters of 0.06 m and 0.07 m. The overall physical structure dimensions of the miniaturized tri-mirror CATR are 0.61 m × 0.2 m × 0.66 m. Simulation results indicate that the cross-polarizations are less than −40 dB along the major cuts of the QZ, with a co-polarization amplitude and phase ripples of 1.6 dB and 10°, respectively. The CATR achieves a QZ utilization ratio of 70%, collectively demonstrating superior QZ characteristics. To further validate the correctness and effectiveness of the design methodology and simulation results, the miniaturized CATR has been fabricated and experimentally tested. Measurement results indicate that at operational frequencies of 183 GHz and 275 GHz, the co-polarization amplitude ripples and phase ripples within a 0.14 m diameter QZ are within 1.8 dB and 11°, respectively. The experimental results exhibit excellent agreement with simulations, confirming the feasibility and validity of the proposed methodology. This approach not only provides a theoretical foundation for achieving high QZ characteristics designs in CATR systems but also offers technical support for constructing a flexible, portable, and miniaturized terahertz CATR system under spatially constrained conditions. These advancements hold significant potential for both academic research and practical engineering applications.

## Figures and Tables

**Table 1:** The key parameter values of the tri-mirror system.

Parameter	*θ*_0_ (°)	*α* (°)	*σ*_1_ (°)	*σ*_2_ (°)	*σ*_3_ (°)	*S*_0_ (m)	*S*_1_ (m)	*S*_2_ (m)	*l* (m)	*f*_3_ (m)
Value	15	15	30	59	44	0.13	0.2	0.68	0.38	0.8

**Table 2:** The simulated QZ characteristics of the developed miniaturized tri-mirror CATR for major cuts (QZ diameter 0.14 m).

Frequency (GHz)	Co-Polar Amplitude Ripple (dB)	Co-Polar Phase Ripple (°)	Cross-Polar Amplitude (dB)
100	1.56	9.49	−44.05
183	1.36	7.45	−44.52
275	0.95	6.39	−44.57
300	0.85	6.31	−43.73
400	0.68	5.66	−43.78
500	0.62	4.14	−44.17

**Table 3:** The measured results of the designed tri-mirror CATR at 183 GHz and 275 GHz. QZ diameter, 0.14 m.

Frequency (GHz)	0° Co-Pol Amp. (dB)	0° Co-Pol Pha. (°)	90° Co-Pol Amp. (dB)	90° Co-Polar Pha. (°)	Cross-Polar Amp. (dB)
183	1.64	23.5	1.55	10.1	−25.3
275	1.03	9.7	0.98	8.6	−32.2

**Table 4:** The QZ characteristics of the developed miniaturized tri-mirror CATR at 183 GHz when fed by feeds with different cross-polarization characteristics.

Cross-Polar of Feed (dB)	Simulation or Measurement	Co-Pol Amp. (dB)	Co-Polar Pha. (°)	Cross-Polar Amp. (dB)
−50	Simulation	1.36	7.45	−44.52
−29	Measurement	1.64	10.1	−25.30
−29	Re-simulation	1.40	8.30	−28.10

**Table 5:** Comparison between the reported miniaturized CATR and the proposed tri-mirror CATR.

Ref.	Upper Fre. Limit (GHz)	QZ Usage (%)	Size of QZ (m × m)	Size of CATR (m × m × m)	Number of Mirrors	Cross-Polar (dB)
[19]	110	50	0.3 × 0.3	2.8 × 1.6 × 2	1	−29
[20]	110	50	0.3 × 0.3	2.1 × 0.96 × 1.85	1	−30
[21]	110	50	0.3 × 0.3	1.8 × 1.6 × 2.5	1	−25
[22]	110	50	0.3 × 0.3	2.5 × 1.2 × 1.6	1	−27
[23]	200	30	0.3 × 0.3	1 × 1 × 1	1	−30
Proposed	500	70	0.14 × 0.14	0.61 × 0.2 × 0.66	3	−40

## Data Availability

The original contributions presented in this study are included in the article. Further inquiries can be directed to the corresponding author.
